# Patterns and predictors of language representation and the influence of epilepsy surgery on language reorganization in children and young adults with focal lesional epilepsy

**DOI:** 10.1371/journal.pone.0238389

**Published:** 2020-09-08

**Authors:** Carmen Barba, Domenico Montanaro, Laura Grisotto, Francesca Frijia, Simona Pellacani, Anna Cavalli, Susanna Rizzi, Matteo Lenge, Gayane Aghakhanyan, Valentina Sibilia, Flavio Giordano, Tiziana Pisano, Francesco Mari, Federico Melani, Andrea Cherubini, Annamaria Buccoliero, Maria Eugenia Caligiuri, Renzo Guerrini

**Affiliations:** 1 Neuroscience Department, Children’s Hospital Meyer-University of Florence, Florence, Italy; 2 U.O.C. RM Specialistica e Neuroradiologia, Fondazione CNR/Regione Toscana G. Monasterio, Pisa, Italy; 3 Department of Statistics, University of Florence, Florence, Italy; 4 Bioengineering and Clinical Technology Unit, Fondazione CNR/Regione Toscana G. Monasterio, Pisa, Italy; 5 Neurosurgery Department, Children’s Hospital Meyer-University of Florence, Florence, Italy; 6 Institute of Molecular Bioimaging and Physiology, National Research Council, Catanzaro, Italy; 7 Pathology Unit, Children’s Hospital Meyer-University of Florence, Florence, Italy; 8 Neuroscience Research Center, University “Magna Graecia”, Catanzaro, Italy; 9 IRCCS Stella Maris, Pisa, Italy; University of Modena and Reggio Emilia, ITALY

## Abstract

Mapping brain functions is crucial for neurosurgical planning in patients with drug-resistant seizures. However, presurgical language mapping using either functional or structural networks can be challenging, especially in children. In fact, most of the evidence on this topic derives from cross-sectional or retrospective studies in adults submitted to anterior temporal lobectomy. In this prospective study, we used fMRI and DTI to explore patterns of language representation, their predictors and impact on cognitive performances in 29 children and young adults (mean age at surgery: 14.6 ± 4.5 years) with focal lesional epilepsy. In 20 of them, we also assessed the influence of epilepsy surgery on language lateralization. All patients were consecutively enrolled at a single epilepsy surgery center between 2009 and 2015 and assessed with preoperative structural and functional 3T brain MRI during three language tasks: Word Generation (WG), Rhyme Generation (RG) and a comprehension task. We also acquired DTI data on arcuate fasciculus in 24 patients. We first assessed patterns of language representation (relationship of activations with the epileptogenic lesion and Laterality Index (LI)) and then hypothesized a causal model to test whether selected clinical variables would influence the patterns of language representation and the ensuing impact of the latter on cognitive performances. Twenty out of 29 patients also underwent postoperative language fMRI. We analyzed possible changes of fMRI and DTI LIs and their clinical predictors. Preoperatively, we found atypical language lateralization in four patients during WG task, in one patient during RG task and in seven patients during the comprehension task. Diffuse interictal EEG abnormalities predicted a more atypical language representation on fMRI (p = 0.012), which in turn correlated with lower attention (p = 0.036) and IQ/GDQ scores (p = 0.014). Postoperative language reorganization implied shifting towards atypical language representation. Abnormal postoperative EEG (p = 0.003) and surgical failures (p = 0.015) were associated with more atypical language lateralization, in turn correlating with worsened fluency. Neither preoperative asymmetry nor postoperative DTI LI changes in the arcuate fasciculus were observed. Focal lesional epilepsy associated with diffuse EEG abnormalities may favor atypical language lateralization and worse cognitive performances, which are potentially reversible after successful surgery.

## Introduction

Patients with drug-resistant epilepsy may benefit from surgical treatment with rates of seizure freedom ranging from 36% to 84% [1]. When seizures arise from, or in close proximity to, eloquent cortical areas, precise preoperative functional mapping is needed to seek complete resection of the seizure onset zone (SOZ), thus increasing the chances for seizure freedom and minimizing the risk for postoperative deficits [2]. However, mapping brain functions using either functional or structural networks can be challenging, especially for language assessment. Although the “classical” model for a language system focuses on Broca and Wernicke areas, more complex models of language representation, including at least six core clinically relevant eloquent areas, have been suggested [3].

According to the recent practice guidelines of the American Academy of Neurology (AAN) [4], fMRI can be considered an option for lateralizing language functions in place of the intracarotid amobarbital procedure (IAP) in adult patients with medial temporal lobe epilepsy, temporal lobe epilepsy in general, or extratemporal epilepsy, but not in those with neocortical temporal epilepsy and tumoral epilepsy. The boundaries and lateralization of language areas may vary depending on the patient’s language skills, the analysis parameters used, the task and control conditions and the eloquent areas to be identified [5]. Cortical mapping is especially challenging in children [5, 6], due to limited cooperation and movement artefacts, forcing the different centers to adopt variable strategies [2]. A few fMRI studies have suggested that results obtained in children and adolescents may appear similar to those observed in adults [4, 7–10]. However, there are no Class I or II studies assessing the diagnostic yield of fMRI in comparison with IAP and other invasive cortical mapping procedures or outcomes in the younger age ranges [4]. Evidence is even poorer for tractography and is mainly related to the evaluation of Meyer's loop prior to temporal lobe surgery [11].

As additional challenge for the surgical planning in the pediatric age resides in the capacity of the child’s brain to significantly reorganize neurologic functions [12]. Developmental brain plasticity compensating for a focal structural brain abnormality may depend on multiple factors, including age of the child and maturational state of the brain at the time of injury, extent, topography and type of the lesion, integrity of cortical areas surrounding or contralateral to it, presence and duration of epilepsy, and AED effects [13]. Whether the developmental versus acquired nature of the lesion may have a driving role in language reorganization is still a matter of debate [9, 13–15].

Owing to the complexity of language organization and uncertainties concerning reliability of the results of cortical mapping, predictors of language laterality have been investigated to a limited extent and generated contrasting findings. As pointed out by several groups [7–10, 14, 16–22], atypical representation of language occurs more frequently in patients with epilepsy than in healthy subjects. Earlier age at brain injury, longer epilepsy duration and atypical handedness have been associated to more frequent atypical lateralization of language in some studies [7, 8, 10, 18, 22] but not in others [9, 14]. Most of the evidence on this topic, however, derives from cross-sectional or retrospective studies in adults submitted to anterior temporal lobectomy. It remains unclear whether observations deriving from these studies also apply to children and adolescents, in whom lesion location and types are quite variable, as are cognitive skills and ability to cooperate with the procedure [5, 23].

In this prospective study, we used fMRI and DTI to explore the possible patterns of language representation (relationship with the epileptogenic lesion and laterality) in 29 children and young adults with focal lesional epilepsy, and analyzed their clinical predictors and effect on cognitive performances. In 20 of the patients, we also assessed the impact of epilepsy surgery on language lateralization and the variables that may influence postoperative changes.

## Materials and methods

### Design of the study and population

This is a prospective study. Patients were consecutively enrolled at Neuroscience Department, Children’s Hospital Meyer, Florence, Italy, between 2009 and 2015. Inclusion criteria were: 1) focal lesional epilepsy, 2) capacity to cooperate for language fMRI studies and 3) planned neurosurgery. Exclusion criteria were: 1) any contraindication to 3T structural and functional MRI, and 2) lack of informed consent.

The experimental protocol is described below; patients were enrolled before undergoing preoperative fMRI.

Thirty-six patients met the inclusion criteria. We excluded from the analysis seven patients (19.4%) for the following reasons: preoperative fMRI protocol not completed (three patients), technical problems affecting preoperative fMRI analysis (two patients) and refusal to undergo postoperative structural 3T MRI (two patients).

For the presurgical study, we enrolled 29 patients (15 F; mean age at surgery: 14.6 ± 4.5 years) whose clinical and demographic information is summarized in Table 1.

**Table 1:** General characteristics, neuroimaging, electroclinical features of 29 patients included in the study.

**Total n˚ Patients**	29
**Sex**	14M/15F
**Age at seizure onset**	7.6± 4.6 y
**Age at surgery**	14.6 ± 4.5 y
**Disease duration**	7 ± 5.0 y
**Pts < 18 yrs**	25 (range 10.2–17.2 y)
**Type of sz**	29 Focal
	11 Focal to bilateral tonic-clonic
**Seizure frequency**	14 Daily
	10 Weekly
	3 Monthly
	2 Yearly
**Preoperative cognitive level**	21 Normal
	5 Mild ID
	3 Moderate ID
**Neurological signs**	6 (2 visual field defects; 2 hemiparesis, 2 UE deficit)
**Handedness**	25 right-handed, 4 left-handed or ambidextrous
**Structural MRI**	25 (Unilobar;19 T), 4 Multilobar
	8 R/ 21 L
**Functional MRI**	29 Language Tasks: • 27 WG task: 14 intralesional, 18 perilesional activation, 4 atypical lateralization • 23 RG task: 10 intralesional, 16 perilesional activation, 1 atypical lateralization • 23 comprehension task: 13 intralesional, 18 perilesional activation, 7 atypical lateralization
**Scalp Interictal EEG**	9 Diffuse or Bilateral
	20 Focal
**Scalp Ictal EEG**	9 Diffuse or Bilateral
	18 Focal
	2 Unavailable
**Invasive recordings**	9/29 patients (31%): 4 grids and 5 SEEG, all before fMRI
**SOZ**	
**Side**	8 R/21 L
**Topography**	23 Unilobar
	6 Multilobar/Hemispheric
**SOZ vs Lesion**	29 SOZ superimposed to lesion as lateralization
	22 SOZ superimposed to lesion as extent
**Histopathology**	FCDI:4
	FCDIIIa: 2
	FCD II: 4
	Tubers: 1
	Tumors: 14 (3 astrocytoma, 5 ganglioglioma, 5 DNET, 1 angiocentric glioma)
	Scars: 4, of which 2 with associated dysplasia (FCDIIId)
**Surgery**	
**Side**	8 R/21 L
**Type of intervention**	19 T lobe surgeries (8 ant T lobectomies, 8 lesionectomy, 3 complete lobectomy)
	4 F lesionectomy
	2 F lobectomy
	4TPO
**Seizure outcome**	16 IA
	5 ID
	1 II
	7 III

^a^ F: female; FCD: focal cortical dysplasia; ID: intellectual disability; L: left; M: male; R: right; RG: rhyme generation; SEEG: stereoelectroencephalography; SOZ; seizure onset zone; T: temporal; UE: upper extremity; WG: word generation; y: years.

For the postsurgical study, we excluded nine patients for the following reasons: a) technical problems affecting postoperative fMRI analysis (five patients); b) parental refusal, as our informed consent procedure allowed withdrawal from the study at any time (four patients). We then assessed 20 (69%) patients in the postsurgical study; the time interval between pre- and postoperative MRIs was 1.7± 1.6 years; the time interval between surgery and postoperative evaluation was 1.3 ±1.4 years.

This study was approved by the Pediatric Ethics Committee on Human Experimentation of the Tuscany Region. Written informed consent was obtained from all patients (or their guardians) participating in the study.

### Presurgical evaluation and surgery (Table 1)

All patients underwent a presurgical evaluation including clinical history, prolonged scalp video-EEG monitoring (>24 h), structural 3T MRI and neuropsychological assessment.

Whenever non-invasive presurgical assessment was inadequate for defining the area to be removed, we performed invasive EEG recordings. We used either stereoelectroencephalography (SEEG) or a combined depth and subdural electrode approach. We identified the SOZ [24] based on non-invasive and, when available, invasive recordings and defined its correlation with the epileptogenic lesion.

We assessed the extent and completeness of the resection of the epileptogenic lesion through postoperative MRI. For histopathology purposes, we used the WHO classification of CNS tumors [25] and the ILAE classification of focal cortical dysplasia (FCD) [26].

We evaluated surgical outcome at last follow-up according to Engel’s classification [27]. All patients underwent postsurgical clinical monitoring including repeat EEGs and neuropsychological evaluations and at least one structural MRI. All 20 patients included in the postoperative study were evaluated with an EEG and neuropsychological testing in the 6 months preceding or following postoperative fMRI.

### Experimental protocol

#### Structural and functional MRI

All patients underwent preoperative structural and functional brain MRI using a 3T scanner (Excite HDx, GE Medical Systems, Milwaukee, WI; eight channels head coils).

Standard diagnostic sequences included: 3D T2-FLAIR, T2*-GRE, T2-FSE and T1-SE. 3D T1-weighted fast-spoiled gradient echo (FSPGR) images of the whole brain (TR = 10.7 ms TE = 4.9 ms, matrix 256x256, flip angle 13°, slice thickness 1mm with no gap, FOV 25.6 cm, voxel resolution [1.0x1.0x1.0] mm^3^, acquisition time 5.60 min). Axial Diffusion Tensor Imaging (DTI) acquisition was performed with a single-shot EPI (Echo Planar Image) applying 30 different gradient directions uniformly distributed on a sphere through electrostatic repulsion with *b-value* = 0–1.000 s/mm^2^ (TR = 16.000 ms, TE = 86 ms; BW = 250 kHz; voxel resolution: [2 x 2 x 2,4] mm^3^; FOV: 25.6 cm; 57 contiguous slices with 2.4 mm of thickness and no gap; acquisition time: 9.36 min).

All patients underwent a BOLD-functional MRI (EPI- GRE, TR/TE 2500/40ms, flip angle 90°, matrix size 128x128, FOV 24.0 cm, in-plane spatial resolution 1.8 mm, slice thickness 4 mm) with a block designed paradigm and 72 phase volumes obtained from 30 contiguous axial slices of 4mm/0gap thickness (acquisition time 193sec). Each period of the blocks (20sec rest - 20sec task) was repeated 4 times, with a final rest phase. The first epoch always lasted 4 phases (13s) more than the followings, not accounted for analysis, necessary to reach a steady-state condition.

The fMRI protocol included a string of three language tasks: Word Generation (WG) [28] and Rhyme Generation (RG) [29] tasks, for the assessment of phonemic fluency, and a comprehension task for the assessment of the semantic fluency [5]. Before starting the fMRI protocol, patients were instructed on the correct execution of each task outside the MR-room. During the WG task, patients wearing MR-compatible headphones covering both ears, listened to a neutral voice that pronounced a single randomly chosen letter every 5 seconds. During this time period, patients had to silently generate as many words they could that began with each listened letter. The RG task was built in the same way as the WG task, but patients were asked to listen to a short word (e.g. cane) and then silently produce a corresponding rhyming word (e.g. pane). For the semantic fluency task, patients were requested to listen to the description of a category of objects and silently think of as many as possible corresponding objects.

We excluded from the analysis fMRI acquisitions affected by head motion distortions and scanner artefacts. For fMRI BOLD data analysis, we used AFNI software [30]. We corrected images for slice acquisition timing differences and registered them to a reference volume in order to minimize movement-related effects. To perform a spatial smoothing operation, we used a three-dimensional Gaussian kernel with 3mm full width half maximum, in order to enhance signal to noise ratio and further reduce residual movement effects. We computed activation maps using a multiple regression analysis incorporating regressors and describing head movements, as obtained by the volume registration step. To model the hemodynamic response, we used a gamma variate function convolved with a boxcar wave function describing the temporal paradigm and evaluated the activation maps on the basis of a partial F-statistics and a cut-off at p<0.05 uncorrected [31]. As these maps could be affected by patients’ head movements and by global SNR variations, to be sure to select correct functional activated voxels, we visually analyzed the time course of each activated voxel to confirm the fitting with the applied paradigm.

Owing to the clinical characteristics of our sample (i.e. mainly children and overall a poorly collaborative cohort) we applied a subject-specific thresholding to the analysis of fMRI data (p values varied between 0.001 and 0.0001) in order to obtain BOLD maps as much as possible corresponding to the actual functional performance.

Twenty patients were included in the postoperative fMRI study, using the same protocol as for the preoperative mapping (the full MRI dataset is available as supporting information).

#### FMRI laterality index (LI)

In the subject-specific thresholded fMRI maps, we first selected, for each task of the individual patients, only voxels that showed a visual correlation with the applied time course in order to avoid the inclusion of voxels of pseudo-activations caused by noise or movement artefacts that the software hadn’t eliminated. Then, we grouped the selected voxels in four macro—regions of interest (ROIs), on the anatomical basis of simple theoretical functional language patterns: left and right anterior frontal ROIs to delineate the Broca’s area and the frontal dorsolateral region, and left and right posterior temporo-parietal-occipital ROIs to include the Wernicke’s area.

To compute the number of activated voxels in each ROI we used an automated post-processing script with AFNI software [32], according to the methodology described by Ruff and colleagues [33]. We calculated two types of Laterality Indexes, as follows: 1) an hemispheric index, globally comparing all the activated voxels of the left side (L) with those of the right side (R); 2) a functional-ROI index, comparing each functional ROI of L with those of R. To compute LIs, we applied the formula [L—R]/[L + R]. The values ranged from -1 to +1, with -1 indicating complete right hemisphere language dominance and +1 indicating complete left hemisphere dominance. Intermediate values reflected varying degrees of laterality. We set the LI value for defining atypical language lateralization at 0.20 (LI < 0.20 as atypical), according to literature [7, 19].

#### DTI

Twenty-four (82%) out of 29 patients underwent also DTI analysis on arcuate fasciculus.

Diffusion MRI was processed using FSL tools. Head motion and image distortions induced by eddy currents in diffusion data were corrected using FSL’s eddy tool [34]. After distortion correction, DTI data were averaged and concatenated. Bayesian Estimation of Diffusion Parameters Obtained using Sampling Techniques was performed on diffusion data using the bedpostX tool of FSL [35, 36], which allows modelling of crossing fibers within each voxel of the brain. Subsequently, the probtrackX tool of FSL was used to perform tractography of left and right arcuate fasciculi for each subject. Regions of interest (ROI) were manually placed in the language areas that are directly connected by the long segment of the arcuate fasciculus [37]. Due to the variability in the location and size of pre-operative lesion/post-operative scar in the brains of our patients, in order to limit the reconstruction of spurious connections we chose to perform tractography using three anatomical ROIs instead of one or two as in the work by Catani and coworkers [37]. Fractional Anisotropy (FA) maps in the subject’s diffusion space, color-coded according to the principal diffusion direction V1, were used to draw ROIs in the posterior part of superior temporal gyrus (Wernicke’s area) and in the frontal lobe white matter (pars opercularis and triangularis, corresponding to Broca’s area). These two ROIs were defined as “seed” and “target” for tractography. Moreover, a “waypoint” mask was placed in the white matter underlying the inferior parietal cortex (Geschwind’s area). ROI tracing was performed by two neuroimaging experts blinded to clinical diagnosis. Reproducibility of ROI placement was ensured by careful visual inspection of the images.

To account for volume differences, the resulting tracts were normalized by the corresponding ‘waytotal’ value (*i*.*e*., the total number of streamlines surviving the tractography constraints, such as seed, target and waypoint masks), and a probability threshold of 0.01 was set to decrease the risk of erroneous findings due to spurious reconstructions. Finally, tracts were binarized.

After reconstruction of the arcuate fasciculus, we computed the volume of each tract mask and the histogram of the FA values in this mask for each side, normalized by the total number of voxels of the tract. Finally, we calculated the following histogram-derived measures:

- mean, median and mode (measures of central tendency);
- standard deviation (measure of variability);
- skewness (measure of asymmetry);
- kurtosis (can indicate the presence of outliers).

For each of the above-mentioned metrics (i.e., volume of the tract and histogram-derived measures), we calculated a laterality index according to the following formula:

$$\frac{Left\;side\;value-Right\;side\;value}{Left\;side\;value+Right\;side\;value}$$

We set a cut-off value of 0.2 for determining the presence of asymmetry, based on previous reports [38, 39].

For the 20 patients who underwent postoperative functional MRI during language tasks, we calculated volume and FA changes of the arcuate fasciculus after surgery according to the following formula:

$$\frac{M\;post-M\;pre}{M\;pre}$$

with M being either volume or FA. Positive results indicated an increase of FA after surgery, while negative values indicated a decrease of FA after surgery. Pre- and post-operative values of the metrics were compared using paired t-tests with Bonferroni correction and statistical threshold at 0.05.

Finally, we defined whether language lateralization assessed through fMRI was related to possible asymmetries of the arcuate fasciculus in individual patients.

#### Neuropsychology

We tested general cognitive abilities using standardized tools: GMDS-ER [40], Italian versions of the Wechsler Scales [41, 42] and measured non-verbal function using the Leiter-r Scale [43]. Since a limited population and its peculiar clinical features do not permit maximum homogeneity in a cognitive scale, we used a single psychometric measure (IQ/General Developmental Quotient). For the statistical analysis, we labeled patients as 0.1 or 2 respectively if they had normal-to-borderline cognitive development (IQ<70), mild delay (IQ 50–70) or moderate and severe delay (IQ<50).

We evaluated language function using tasks of naming and fluency and assessed the ability to name drawings of objects using the Boston Naming Test [44]. To assess verbal and semantic fluency, we used the word fluency test [45]. We classified language function performances in two categories: normal or pathological based on naming and fluency test scores.

To assess visual attention, each child was asked to perform a task of the barrage of target numbers among the target distractors. To measure the processing speed, we used a task from Wechsler scales (Digit Symbol) [41, 42]. As part of the assessment of verbal and working memory, we used two tasks as follows: digit span forward (SPANFW) and digit span backwards (SPANBW) derived from the Wechsler scales [41, 42].

Improvement, stability or deterioration of cognitive and language functions were defined based on possible changes of category according to the postoperative neuropsychology test scores.

### Statistical analysis

#### Preoperative study

For the purposes of the preoperative study, we hypothesized a causal model to test whether several clinical variables (possible predictors) would influence fMRI results (outcomes) and the latter (possible predictors) would in turn impact on cognitive performances (outcomes).

A causal model is a conceptual model that describes the causal mechanisms of a system, in this case the causal mechanisms of language reorganization in children and young adults with focal lesional epilepsy. The causal model provides clear rules for defining what independent variables need to be included or controlled for and what models to adopt [46]. Since the causal model defines the structure of the statistical analysis, we have not evaluated indirect associations and hierarchical structures of the data and models other than those defined by the casual model.

For each patient, we then analyzed the following three groups of variables, as follows:

- Clinical features:
  - General characteristics: gender, handedness, neurological deficits, age at seizure onset, seizure frequency, age at surgery, duration of epilepsy.
  - Neuroimaging: localization, lateralization and extent (uni- or multilobar) of the epileptogenic lesion.
  - Neurophysiology: topography of ictal and interictal EEG abnormalities (focal vs diffuse epileptiform activity i.e. spikes, polyspikes, spike and waves, sharp waves; ipsilateral vs contralateral epileptiform activity with respect to the epileptogenic lesion), extent of the SOZ (unilobar vs multilobar) and its relationship with the structural abnormality (whether superimposed to the lesion or extending beyond it).
  - Surgical variables: Engel’s class, histopathology.
- FMRI results:
  - Intralesional vs perilesional activation and ipsilateral vs contralateral activation (with respect to the epileptogenic lesion) during each task. We classified activated voxels as “intralesional”, when included in the MRI visible structural abnormality, and “perilesional” when located in the apparently structurally normal brain bordering the lesion. When the limits of the structural abnormality were ill-defined at MRI (mainly in FCD type I), we anatomically referred to the surgical breach, providing that histopathology confirmed residual abnormal tissue bordering surgical excision margins.
  - Global LI for each language task.
- Neuropsychological findings:
  - IQ (Intelligence Quotient) or GDQ (General Developmental Quotient) scores.
  - Phonemic fluency and naming (pathological vs normal).
  - Attention (pathological vs normal).
  - Forward and backward span (pathological vs normal).

We defined a clinical cut off for the discretization of continuous variables wherever possible, because the discretization improves the efficiency of estimates when only a limited number of observations is available [47].

Owing to the high number of parameters of interest, we first performed an exploratory data analysis for each group through a factorial analysis for mixed data (FAMD) [48], in order to analyze the relationship between the individual parameters and select variables to be used as possible predictors in the subsequent analysis of associations [48]. FAMD is a principal component method allowing to explore the association between quantitative and qualitative variables and analyze the similarity between individuals in order to reduce a high number of variables in a few latent ones.

Then, for the analysis of associations, we used Pearson’s χ^2^ test of independence and, if necessary, the exact test of Fischer, Student’s t-test and ANOVA (analysis of variance). To estimate the effect of each parameter on outcomes, we used generalized linear regression models.

We selected the covariates for the univariate analysis through the exploratory analysis. Owing to the small sample size, we did not consider possible confounders such as handedness in the univariate analysis, but we only estimated crude effects. If these effects were significant (p-value of regression coefficient < 0.05), we then computed multivariate models. For univariate and multivariate models, we used likelihood ratio test for overall model evaluation ad adjusted R^2^ or pseudo R^2^ as goodness of fit measures. For multivariate models, we carried out a sensitivity analysis for each model using a stepwise method and specified the significance level as addition to the model at 0.05; terms with p < 0.05 were eligible for addition. When an estimation was not possible in the univariate model i.e. GLR estimates had standard errors questionable or the confidence intervals cannot be estimated, we reported the exact test results. Given the exploratory nature of the study we did not perform any correction for multiple comparisons.

We carried out all statistical analyses using STATA version 13.0 (StataCorp. 2013. Stata Statistical Software: Release 13. College Station, TX: StataCorp LP) and R version 3.3.3 (2017-03-06) Copyright © 2017 The R Foundation for Statistical Computing.

#### Postoperative study

In the 20 patients who underwent postoperative fMRI during language tasks, we computed LI changes after surgery (labelled as Delta-LIs). We performed an analysis of associations between Delta-LIs and the following clinical variables, which were chosen based on previous studies on the topic [49–54]: lesion lateralization (right vs left); postoperative scalp interictal EEG (normal vs pathological); histology (FCD, tumors, other); seizure outcome (Engel class IA vs Engel class IB-IV); disease and follow-up duration; IQ/GDQ scores, fluency and naming (improved, worsened, stable).

## Results

### Preoperative study

#### Functional MRI (Figs 1 and S1)

**Fig 1:**
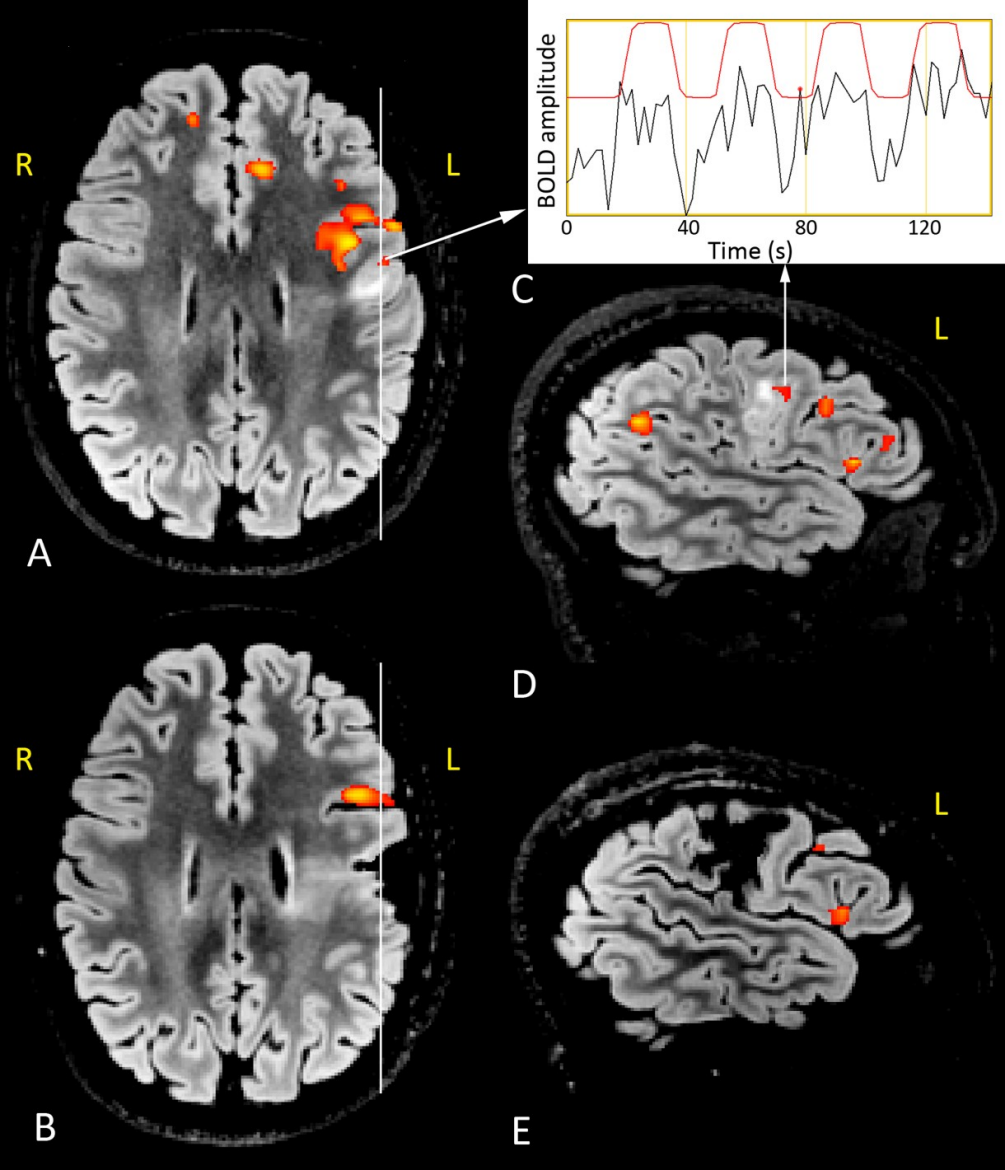
Presurgical and postoperative 3T structural and functional MRI during WG task. Right-handed 15-year-old girl with left frontal FCD IIb and left dominance for language. BOLD functional correlated signals (red-yellow colormap) are superimposed on axial FLAIR images (A, presurgical; B, postsurgical) and sagittal FLAIR sections (D, presurgical; E, postsurgical) across the lesion and the surgical breach (see white vertical lines). Structural MRI shows a left frontal malformed area located on a irregularly flat gyrus whose subcortical white matter is mostly hyperintense. The time course of the BOLD signal of the area indicated in the preoperative images is showed in C (red line, time course of the task; black line, the variation of the BOLD signal during the task. The red line indicates the GLM model of HRF response and the black one the raw signal registered during the acquisition). In the presurgical examinations (A, D), the cluster of BOLD pixels located inside the lesion (white arrows from A and D to C) correctly correlates with the fMRI paradigm. This cluster, labelled as ‘intralesional’, is no longer evident in the postoperative fMRI study due to the removal of the dysplastic area. The frontal BOLD correlated functional clusters, located in the normal brain bordering the anterior aspect of the lesion in pre and postsurgical examinations, are labelled as ‘perilesional’. Right and left sides are indicated in yellow.

Structural and functional MRI findings are summarized in Table 1.

Among the 29 patients who underwent fMRI study, twenty-seven patients (93%) performed the WG task, 23 (79%) the RG task and 23 (79%) the comprehension task. Twenty-one (72%) patients underwent all three tasks.

The average Global LI was 0.47 (SD 0.48) for WG task, 0.56 (SD 0.33) for RG task and 0.33 (SD 0.56) for comprehension task. The distribution of atypical lateralization (LI < 0.20) and crossed dominance (dissociation between phonological and syntactic-semantic aspects of language) across the different tasks is summarized in Table 1.

**Fig 2:**
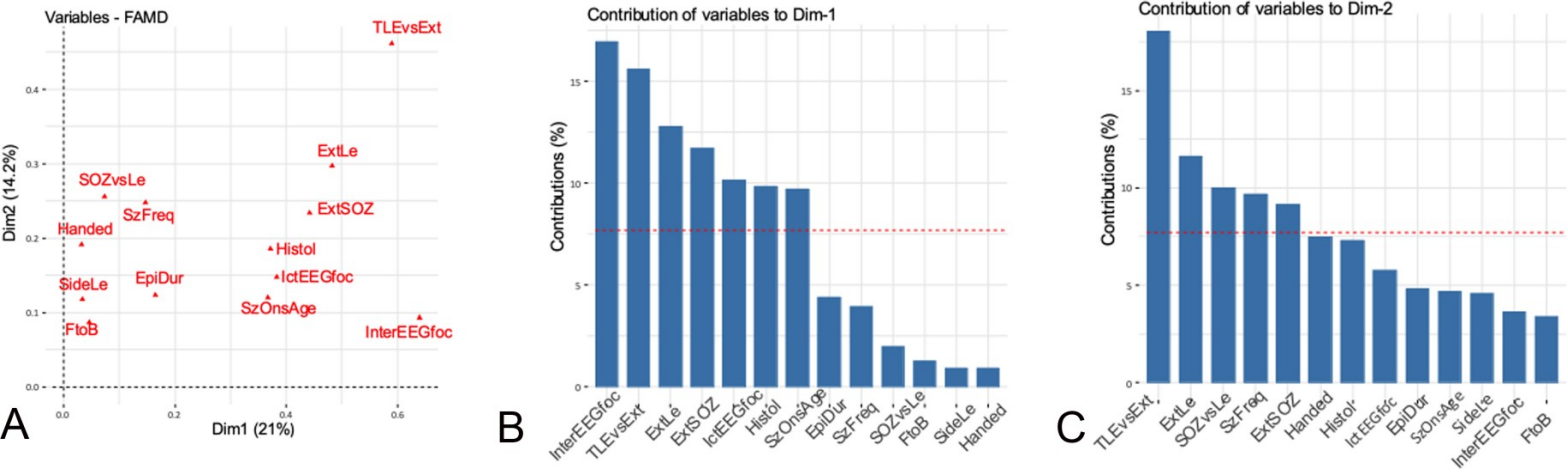
Clinical variables. Correlation between quantitative and qualitative variables and the principal dimensions (A) and contribution of the variables to dimensions 1 (B) and 2 (C). The red dashed line on the graph indicates the expected average values, if the contributions were uniform. ^a^EpiDur: Epilepsy duration; ExT: extratemporal; Foc: focal; FtoB: focal to bilateral tonic-clonic; Handed: handedness; IctEEG: Ictal EEG, InterEEG: interictal EEG; Le: lesion; SOZ: seizure onset zone; SzOnAge: age at seizure onset; TLE: temporal lobe epilepsy; VS: versus.

**Fig 3:**
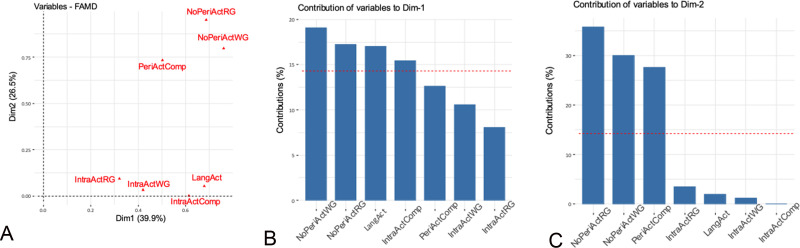
Functional MRI variables. Correlation between quantitative and qualitative variables and the principal dimensions (A) and contribution of the variables to dimensions 1 (B) and 2 (C). The red dashed line on the graph indicates the expected average value, if the contributions were uniform. ^a^ IntraActComp: intralesional activation during comprehension task; IntraActRG: intralesional activation during RG task; IntraActWG: intralesional activation during WG task; LangAct: fMRI activation during all language tasks; NoPeriActRG: absence of perilesional fMRI activation during RG task; NoPeriActWG: absence of perilesional fMRI activation during WG task; PeriActComp: perilesional fMRI activation during comprehension task.

**Fig 4:**
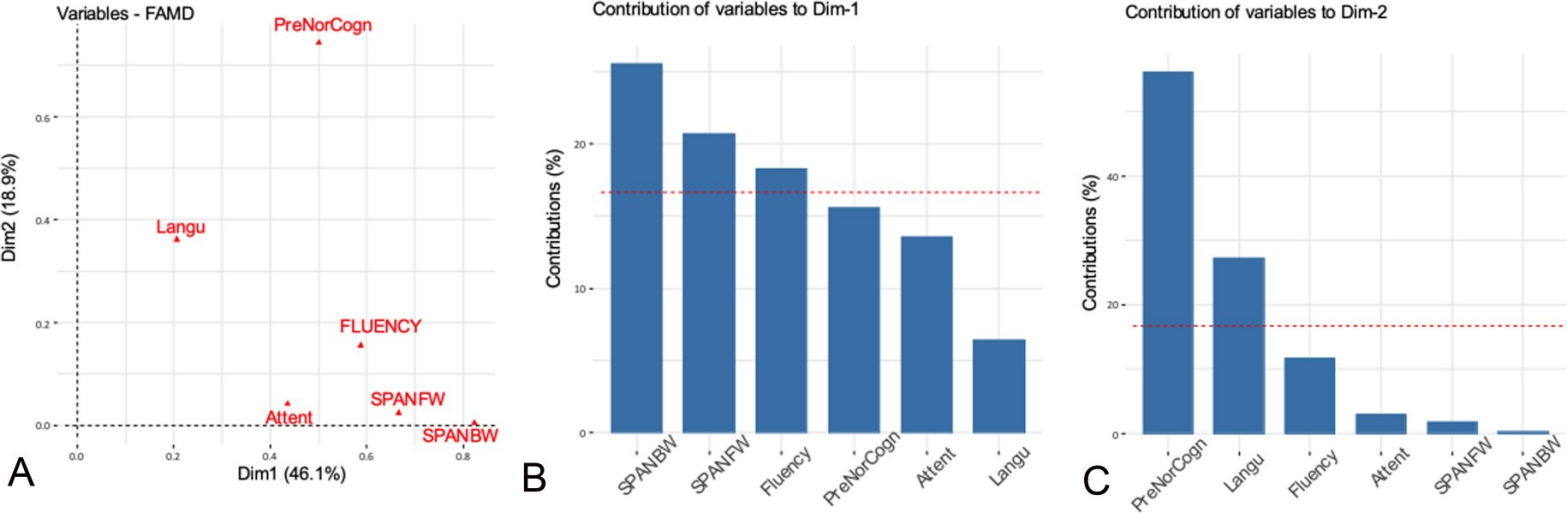
Neuropsychological variables. Correlation between quantitative and qualitative variables and the principal dimensions (A) and contribution of the variables to dimensions 1 (B) and 2 (C). The red dashed line on the graph indicates the expected average value, if the contributions were uniform. ^a^Attent: attention scores; Langu: language scores: PreNorCogn: preoperative normal cognitive scores; SPANBW: backward span scores; SPANFW: forward span scores.

According to the exploratory analysis (Figs 2–4 and S1 Table), we selected as possible predictors for the analysis of associations the variables significantly correlated with the first two dimensions of the FAMD, as follows: a) *clinical variables—*interictal and ictal scalp EEG, topography of the lesion and the SOZ (side and localization) and their relationship, histology, handedness, epilepsy duration, age at seizure onset, seizure frequency, focal to bilateral tonic-clonic seizures; b) *fMRI findings*—LIs and intra- and perilesional activations during all language tasks; c) *neuropsychological findings*—global cognitive abilities, fluency, language and backward and forward span and attention scores.

We then assessed through univariate analysis (see S2 Table), according to our causal model, whether any of the selected clinical variables may represent a predictor of the fMRI results, thereby disclosing the following significant associations:

- Patients with diffuse or bilateral ictal scalp EEG had a 100% probability of exhibiting an intralesional activation during all language tasks, while patients with focal ipsilateral ictal EEG had 50% probability (Fisher’s exact test, p = 0.012).
- Patients with diffuse or bilateral ictal scalp EEG had a higher probability of disclosing an intralesional or perilesional activation during WG task than patients with focal ipsilateral ictal scalp EEG (OR = 12.83, p = 0.031, LR χ^2^_(1)_ = 6.51 p = 0.011, Pseudo R^2^ = 0.19 and OR = 6.27, p = 0.05, LR χ^2^_(1)_ = 4.47 p = 0.034, Pseudo R^2^ = 0.09, respectively).
- Patients with intralesional activation during RG task had a higher average duration of epilepsy (OR = 1.25, p = 0.049, LR χ^2^_(1)_ = 5.65 p = 0.017, Pseudo R^2^ = 0.17).

The multivariate linear regression model revealed a significant effect of diffuse interictal scalp EEG abnormalities (regr. coeff = -0.401 p = 0.014) and handedness (regr. coeff = -0.768 p = 0.002) on Lis (Table 2). Specifically, language lateralization was more atypical if EEG abnormalities were bilateral or diffuse rather than focal or unilateral and if patients were left-handed rather than right-handed.

**Table 2:** Multivariate linear regression model of LIs.

Predictor		β	SE β	Wald test	p-value
Constant		1.841	0.274	45.25	<0.001
Diffuse interictal scalp EEG abnormalities		-0.401	0.151	12.24	0.014
Handedness		-0.768	0.220	7.06	0.002
Overall model evaluation:					
	Likelihood ratio test χ^2^_(2)_ = 20.270 p<0.001				
Goodness of Fit:					
	Adj R^2^ = 0.489				

The likelihood ratio test was significant (LR χ^2^_(2)_ = 20.270 p<0.001), thus indicating that our model is more effective than the null model. The proportion of the total variability explained by the model as calculated by the adjusted R^2^ was 48.9%. The sensitivity analysis confirmed the results of the multivariate regression model.

Regression models showed that LI during comprehension task became more atypical when age at seizure onset increased (regr. coeff = -0.051; p = 0.036, LR χ^2^_(1)_ = 4.93 p = 0.026, Adj R^2^ = 0.15) and if patients were left-handed (regr. coeff = -0.6; p = 0.020, LR χ^2^_(1)_ = 6.02 p = 0.014, Adj R^2^ = 0.19).

Conversely, side and localization of the lesion and of the SOZ, histology, epilepsy duration, age at surgery and seizure frequency did not predict fMRI results.

As a further test included in our causal model, we evaluated, through univariate analysis, whether the pattern of language representation had an impact on cognitive performances, thereby disclosing the following significant associations:

- Patients with intralesional activation during all language tasks had a higher probability of exhibiting a pathological backward span (Fisher’s exact test, p = 0.023).
- Patients with severe cognitive impairment disclosed a more atypical language lateralization (p = 0.0136) compared to those with higher cognitive scores.
- Patients with lower attention scores had a more atypical language lateralization during WG and RG (F = 21.23, p<0.001 and F = 5.10 p = 0.036, respectively) and comprehension tasks (F = 4.73, p = 0.043) compared to those with higher attention scores.

Univariate and multivariate models did not show any significant result.

#### DTI

The mean Volume LI for the arcuate fasciculus was -0.071 and the mean FA LI was -0.022.

Histogram analysis did not highlight hemispheric differences in the average distribution of FA values extracted from the arcuate fasciculus. The calculation of arcuate fasciculus LIs for volume and histogram-based measures could not identify any strong asymmetric pattern. LI values rarely exceeded 0.2 for left asymmetry (-0.2 for right asymmetry) (S2 Fig)

We found no correlation between fMRI language dominance and arcuate fasciculus LI values in most patients.

### Postoperative data analysis

#### FMRI (Tables 3 and S3)

Comparing pre- and postoperative LI values, we found atypical language lateralization in one vs three patients during WG task, in none vs three patients during RG task, in two vs seven patients during comprehension task. The analysis of variations of preoperative vs postoperative LIs (paired Student’s t-test) is summarized in Table 3. All changes determined a more atypical language representation after surgery compared to preoperative measures. In particular, we found significant LI changes for RG (anterior frontal ROI) and comprehension tasks (global and anterior frontal ROIs).

**Table 3:** Average (±sd) of preoperative and postoperative LIs for global, temporal and fronto-dorsal ROIs. P-value of paired Student’s t-test.

		N° of Patients	Pre LI ±sd	Post LI ±sd	P-value
LI-WG					
	Global	20	0.62 ±0.24	0.52 ±0.45	0.318
	Temporal	17	0.49 ±0.59	0.44 ±0.63	0.797
	Fronto-dorsal	20	0.73 ±0.22	0.55 ±0.40	0.098
LI-RG					
	Global	16	0.59 ±0.22	0.40 ±0.40	0.061
	Temporal	15	0.55 ±0.36	0.50 ±0.53	0.698
	Fronto-dorsal	16	0.65 ±0.25	0.41 ±0.37	0.032
LI-Comp					
	Global	15	0.53 ±0.37	0.24 ±0.39	0.007
	Temporal	14	0.65 ±0.50	0.46 ±0.56	0.165
	Fronto-dorsal	15	0.54 ±0.42	0.22 ±0.37	0.004

^a^Comp: comprehension; LI: laterality Index; N: number; Pre: preoperative; Post: Postoperative; RG: rhyme generation; ROI: region of interest; WG: word generation.

When analyzing possible clinical predictors of postoperative changes of LI through univariate analysis (S2 Table), we found that abnormal postoperative interictal scalp EEG (p = 0.003), Engel class IB-IV outcome (p = 0.015) and a longer disease duration were significantly associated with a more atypical language representation after surgery. This latter was in turn associated with a worsened fluency after surgery compared to preoperative performances.

In addition, in patients with right epileptogenic lesions, atypical language representation was observed more often than in those with left epileptogenic lesions (mean ±std -0.65±0.46 vs -0.14±0.35; p = 0.049).

Multivariate analysis did not disclose significant results.

#### DTI

For the arcuate fasciculus, the mean postoperative Volume LI was-0.037 and the mean postoperative FA LI was -0.018.

We identified no significant postoperative FA and volume LI changes.

## Discussion

The main aim of this prospective study was to explore the possible patterns of representation of cortical language function and analyze their clinical predictors in 29 children and young adults with focal lesional epilepsy.

The average global LIs for all tasks indicated an overall typical lateralization of language, a finding which is at odds with previous studies that suggest a more frequent atypical lateralization of language in patients with epilepsy [7–10, 14, 16–22]. Many differences in inclusion criteria and study protocols make difficult the comparison between our own and previous series hampered.

The factorial analysis for mixed data highlighted the preeminence of scalp EEG findings among clinical predictors of language lateralization. This finding was supported by univariate and multivariate regression analyses showing a correlation between diffuse scalp EEG abnormalities and more atypical language representation. Janszky and colleagues [20, 21] found that atypical speech representation was associated with higher spiking frequency in adults with left TLE and suggested that chronic frequent interictal activity might induce a reorganization of speech lateralization. Liegeois et al [9] could not confirm these findings in ten children with epilepsy and early left hemispheric lesions, possibly due to differences in the clinical characteristics of the sample. Our results expand these previous observations by indicating that diffuse scalp EEG abnormalities predict a more atypical representation of language, irrespective from the extent, topography and histopathological features of the epileptogenic lesion. In agreement with some studies [7, 8, 10, 18, 22], but at odds with others [9, 14], side and localization of the lesion and of the SOZ, epilepsy duration, age at surgery, seizure frequency and histology did not predict fMRI results. Heterogeneity of the sample, however, is always a factor that might influence results, in ours as in other series.

Our preoperative findings are sustained by the results of the longitudinal language fMRI study which demonstrated significant postoperative LI changes for RG and comprehension tasks, thus suggesting a reorganization of language after surgery. A longitudinal evaluation of language reorganization after epilepsy surgery was reported in six previous small-sample studies [49–54], of which four [49, 50, 52, 53] described intra and interhemispheric reorganization after temporal lobe surgery in adults. In particular, Helmstaedter et al. [52] described a patient with left temporo-mesial epilepsy in which language dominance shifted from the right to the left hemisphere after successful epilepsy surgery. The only two pediatric longitudinal fMRI observations [51, 54] concerned individual cases with Rasmussen syndrome in whom language-related networks shifted to the right hemisphere after left hemispherectomy. Our finding that postoperative EEG (p = 0.003) and seizure outcome (p = 0.015) were predictors of language reorganization after surgery, confirms that, at least in children and young adults, atypical language dominance can be functionally driven, shifting back to a more typical representation when the driving factor i.e. interictal EEG abnormalities and seizures, is controlled. This finding seems to hold across different localizations and types of epileptogenic lesions.

As additional finding, patients with diffuse or bilateral ictal EEG discharges had a higher probability to disclose an intralesional or perilesional fMRI activation during language tasks than those with focal ictal EEG discharges. Several factors might have contributed to this result. For instance, previous EEG-fMRI studies [55, 56] have shown widespread and bilateral spikes to be more frequently associated with BOLD-responses than focal spikes, irrespective of the pathologic substrate, with most patients disclosing fMRI activation near or inside the epileptogenic lesion [57]. Histopathology might also have a role, since in our study most patients with intralesional activation and diffuse ictal EEG harbored scars or FCD type I, which are usually associated with ill-defined borders and widespread epileptogenicity [58, 59]. The observation of BOLD activated areas in the context of inhomogeneous lesions such as scars and tumors is difficult to interpret from the pathophysiological point of view. We can rule out the possibility that these activations are artefactual, since interferences from head movements were corrected by the post-processing analysis and the time variation courses of the BOLD signal were carefully assessed with respect to noise. On the other hand, most tumors in our sample can be classified as "developmental tumors [58]" and in two of the three patients harboring scars these were part of FCD IIId [26]. Associated malformation components may still carry out eloquent functions and correlate with the intralesional BOLD activations we found [60]. Also, the discrepancy in spatial resolution between BOLD MRI and anatomical sequences (matrix size 128x128 vs 512x512) prevented us from demonstrating the possible presence of normal brain tissue in the context of the epileptogenic lesion.

Our study of associations indicates age at seizure onset as a weaker predictor of preoperative language lateralization on fMRI, an observation which is in line with several previous studies [7, 8, 18], but at odds with others [9, 21, 22]. In addition, we observed that longer disease duration was associated with more atypical language lateralization after surgery. However, the influence of epilepsy duration on changes of language dominance over time should be interpreted with caution, since a trend towards more lateralized language representation with increased age is a phenomenon observed in healthy children [61].

Rather unexpectedly, we found only a weak correlation between the side of lesion and of its surgical removal and postoperative LI changes. This finding might be due to the high percentage of small lesionectomies and anterior temporal lobectomies [62] and the low percentage of non-dominant resections in our sample [63].

A secondary aim of our study was to assess whether an atypical representation of cortical functions may influence cognitive performances. We found a more atypical language lateralization to be associated with lower attention and IQ/GDQ scores prior to surgery and to worsened fluency postoperatively. These findings confirm previous observations [8, 64] about a correlation between atypical representation of language and worse verbal abilities and intellectual skills in patients with epilepsy and expand them to a pediatric population. EEG features might have played a role, since diffuse electrographic abnormalities were associated with both more atypical language lateralization and worse cognitive performances. A role of widespread EEG abnormalities in cognitive dysfunction has already been postulated [65, 66].

The analysis of our DTI data did not reveal preoperative asymmetry in the arcuate fasciculus or a correlation with language functional dominance. Previous studies in healthy subjects [38] and in patients with TLE [39] had described an overall leftward asymmetry of the arcuate fasciculus, often correlating with functional hemispheric language lateralization. It might be hypothesized that lack of preoperative asymmetry in the arcuate fasciculus represents an example of structural reorganization. However, our limited sample size and the single timepoint assessment prevent from drawing any firm conclusion on this point.

Differently from previous postoperative DTI studies investigating the effects of surgery on different brain tracts [67–69], we found no significant postoperative arcuate fasciculus changes. Yogarajah and colleagues [70] suggested that increased FA in the language network may represent a structural reorganization after anterior temporal lobe resection in temporal lobe epilepsy. We believe that the lack of significant postoperative LI changes in our study, compared to previous reports, may be due to different sources of variability, such as the heterogeneity of the sample, the different methods used to analyze MRI data and the regions of interest we investigated. In particular, no previous study specifically evaluated arcuate fasciculus LI changes after surgery. We used tractography to delineate the tract at the subject level avoiding the use of a common space, which would have led to unreliable results due to the large differences in the location of the pre-operative lesions and corresponding surgical breaches. In addition, in order to investigate the brain structure-function relationship, we focused on the arcuate fasciculus to combine fMRI LI analysis of language lateralization to some measures of structural asymmetry. Powell and colleagues [71] found that, in healthy adults, functional and structural lateralization, measured as brain activation and FA, respectively, were directly correlated and tract volume was greater in the left hemisphere. The discrepancy of our results from those of Powell’s study [71] may be partly due to the different sample characteristics, particularly healthy adults vs a pediatric cohort of operated epilepsy patients. Sample heterogeneity, variability of postoperative fMRI timepoints and lack of normative data limit the consequences of our study. In addition, our focusing on Broca and Wernicke areas can be simplistic, in view of the complexity of language system representation [72]. However, our sample was heterogeneous in etiology and mainly composed of children and inconsistently cooperative patients, who could only be investigated using a few tasks. Although the inclusion criteria we used increased the challenges of the study, they also helped to provide new insights on the more general mechanisms underlying the cortical representation of language functions and its possible postoperative reorganization. In addition, we could expand our results to the pediatric population in which functional plasticity is particularly relevant to the recovery of linguistic competence after an insult or surgery [12].

Performing fMRI scans in a single setting reduced the variability potentially associated to changes in acquisition protocols and analysis parameters. We obviated to the lack of normative data, which is unavoidably imposed by ethics restrictions, by referring to several previous studies [7, 17–19, 22] reporting consistent figures concerning hemispheric language dominance in healthy subjects. Test-retest reliability was not assessed in our study due to the clinical characteristics of the sample i.e. children with drug-resistant epilepsy awaiting surgery. In fact, owing to methodological challenges, a few studies have investigated test-retest reliability of language fMRI, in healthy adults [73] or in children without epilepsy [74]. We cannot then definitely rule out that some kind of measurement error influenced our results.

Our data suggest that topography of EEG abnormalities and epilepsy duration strongly influence language lateralization in patients with focal lesional epilepsy, irrespective from site and type of lesion. In line with this observation, patients with normal EEG and seizure freedom after surgery exhibited a more typical postoperative language representation. Although localized ictal onset on scalp EEG is a well-recognized predictor of favorable seizure outcome [1], several studies have suggested that early surgery can be beneficial also in patients with focal lesions but diffuse epileptogenicity [75–77]. Our observation that focal lesional epilepsy associated with diffuse EEG abnormalities may lead to atypical language lateralization and worse cognitive performances, which are potentially reversible after successful surgery, further supports this view.

## Supporting information

S1 Fig Fifteen-year-old girl operated on for left frontal drug-resistant epilepsy. Preoperative (A) and postoperative (B) 3T axial T2-weighted images. Postoperative MRI (B) shows the resection cavity after the removal of a histologically proven FCD type I. In the preoperative MRI (A), fMRI clusters correlated with a language paradigm of word generation, are represented as colored spots. The yellow arrow indicates a cluster of fMRI activation inside the epileptogenic lesion (‘intralesional’ activation), while the blue arrow indicates a further cluster located in the normal tissue posterior to the margin of the dysplastic area (‘perilesional’ activation). Right and left sides are indicated in yellow.
(TIF)

S2 Fig Distribution of Laterality Indexes (LI) in pre-operative and post-operative DTI studies. Top row shows LIs of Arcuate Volume; second and third rows show indexes extracted from the histogram of FA distribution in the reconstructed fasciculi.
(TIF)

S1 Table Results of factorial analysis for mixed data (FAMD). Variables, P-value of R^2^ for categorical variables and correlation coefficient for numerical variables significantly correlated with the first two dimensions.
(DOCX)

S2 Table Generalized linear models. Univariate analysis.
(DOCX)

S3 Table Postoperative analysis of associations: Significant results. (DOC)

S1 File (DOCX)
