# Supplementary figures and images for: Patterns and predictors of language representation and the influence of epilepsy surgery on language reorganization in children and young adults with focal lesional epilepsy
Source: PLoS One. 2020 Sep 8;15(9):e0238389. doi: 10.1371/journal.pone.0238389 (PMC7478845; doi:10.1371/journal.pone.0238389)

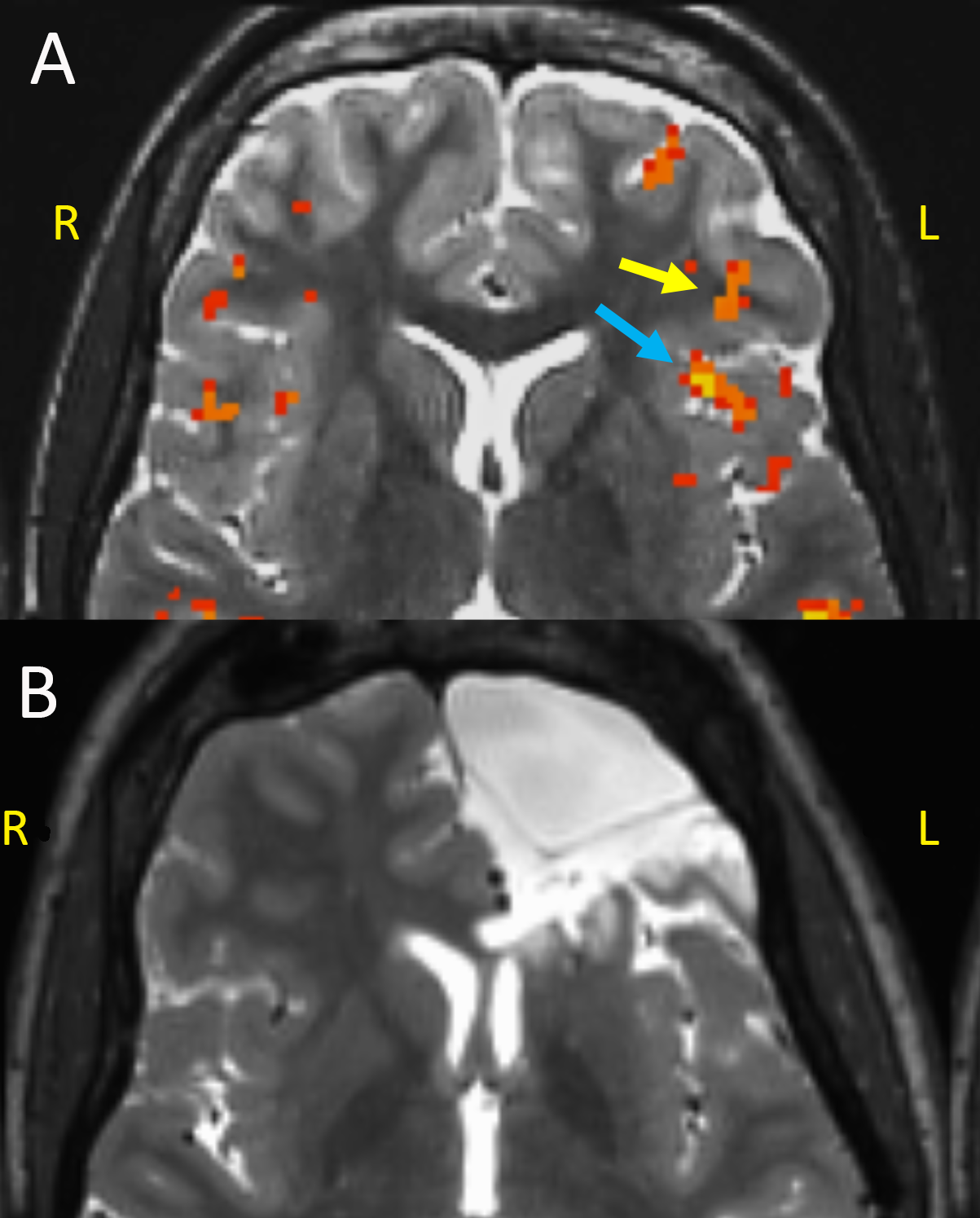

Supplement: S1 Fig — Preoperative (A) and postoperative (B) 3T axial T2-weighted images. Postoperative MRI (B) shows the resection cavity after the removal of a histologically proven FCD type I. In the preoperative MRI (A), fMRI clusters correlated with a language paradigm of word generation, are represented as colored spots. The yellow arrow indicates a cluster of fMRI activation inside the epileptogenic lesion (‘intralesional’ activation), while the blue arrow indicates a further cluster located in the normal tissue posterior to the margin of the dysplastic area (‘perilesional’ activation). Right and left sides are indicated in yellow. (TIF) [file pone.0238389.s001.tif]

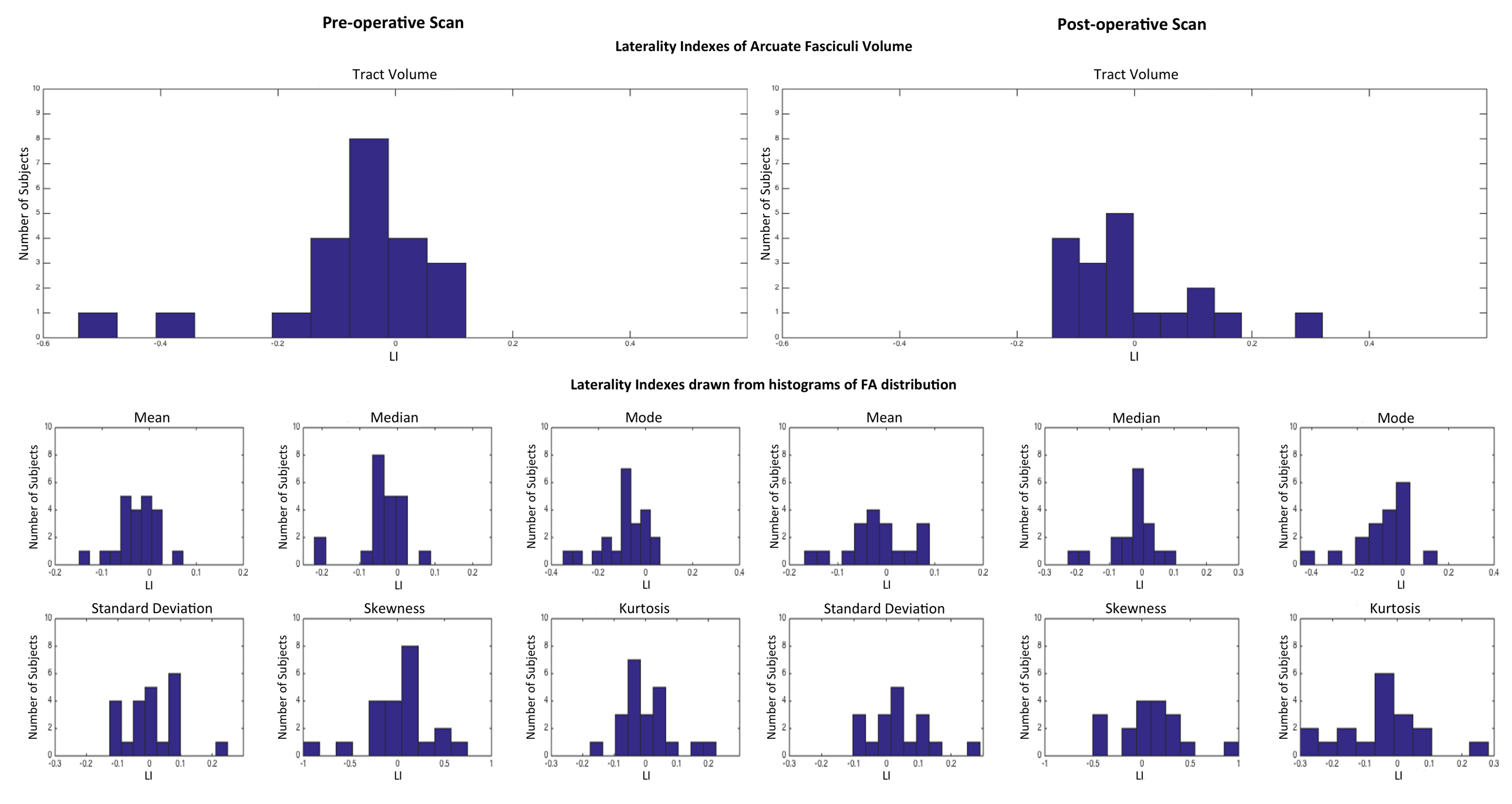

Supplement: S2 Fig — Top row shows LIs of Arcuate Volume; second and third rows show indexes extracted from the histogram of FA distribution in the reconstructed fasciculi. (TIF) [file pone.0238389.s002.tif]
