# Supplementary material for: Patterns and predictors of language representation and the influence of epilepsy surgery on language reorganization in children and young adults with focal lesional epilepsy
Source: PLoS One. 2020 Sep 8;15(9):e0238389. doi: 10.1371/journal.pone.0238389 (PMC7478845; doi:10.1371/journal.pone.0238389)
Supplement: S1 Table — Variables, P-value of R2 for categorical variables and correlation coefficient for numerical variables significantly correlated with the first two dimensions. (DOCX) [file pone.0238389.s003.docx]

**S1 Table: Results of Factorial analysis for mixed data (FAMD)**

| Clinical variables | | | R | Corr | P(value) |
| --- | --- | --- | --- | --- | --- |
|  | first component | | | | |
|  | Epilepsy Duration |  |  | 0.407 | 0.012 |
|  | Age at Seizure Onset | |  | -0.606 | 0.000 |
|  | Focal Interictal EEG | | 0.641 |  | 0.000 |
|  | Temporal lobe epilepsy vs Extratemporal lobe epilepsy | | 0.591 |  | 0.000 |
|  | Extratemporal Lesion |  | 0.484 |  | 0.000 |
|  | Extratemporal  Seizure onset zone |  | 0.443 |  | 0.000 |
|  | Focal Ictal EEG | | 0.385 |  | 0.000 |
|  | Histology |  | 0.373 |  | 0.004 |
|  | Second component | | | | |
|  | Epilepsy Duration |  |  | 0.350 | 0.033 |
|  | Age at Seizure Onset | |  | -0.346 | 0.036 |
|  | Temporal lobe epilepsy vs Extratemporal lobe epilepsy | | 0.461 |  | 0.000 |
|  | Extratemporal Lesion |  | 0.297 |  | 0.000 |
|  | SOZ vs Lesion |  | 0.255 |  | 0.001 |
|  | Extemporal Seizure Onset Zone |  | 0.233 |  | 0.002 |
|  | Handedness |  | 0.191 |  | 0.007 |
|  | Seizure Frequency |  | 0.247 |  | 0.008 |
|  | Focal Ictal EEG | | 0.147 |  | 0.019 |
|  | Side of Lesion |  | 0.117 |  | 0.038 |
| FMRI findings | | | R | Corr | P(value) |
|  | first component | | | | |
|  | No Perilesional Activation during WG task | | 0.761 |  | 0.000 |
|  | Activation during all Language tasks |  | 0.679 |  | 0.000 |
|  | Intralesional  Activation during Comprehension task | | 0.615 |  | 0.000 |
|  | No Perilesional Activation during RG task | | 0.688 |  | 0.000 |
|  | Intralesional Activation during WG task | | 0.422 |  | 0.002 |
|  | Perilesional Activation during Comprehension task | | 0.503 |  | 0.003 |
|  | Intralesional Activation during RG task | | 0.322 |  | 0.009 |
|  | second component | | | | |
|  | No Perilesional Activation during RG task | | 0.949 |  | 0.000 |
|  | No Perilesional Activation WG task | | 0.796 |  | 0.000 |
|  | Perilesional Activation during Comprehension task | | 0.731 |  | 0.000 |
| Neuropsychological findings | | | R | Corr | P(value) |
|  | first component | | | | |
|  | Backward SPAN | |  | 0.908 | 0.000 |
|  | Forward SPAN | |  | 0.817 | 0.000 |
|  | Fluency |  | 0.589 |  | 0.000 |
|  | Attention scores |  | 0.437 |  | 0.001 |
|  | Preoperative normal Cognitive scores | | 0.502 |  | 0.002 |
|  | Language |  | 0.207 |  | 0.038 |
|  | second component | | | | |
|  | Preoperative normal Cognitive scores | | 0.743 |  | 0.000 |
|  | Language |  | 0.360 |  | 0.004 |

Variables P-value of R^2^ for categorical variables, and correlation coefficient for numerical variables. significantly correlated with the first two dimensions.
