# Supplementary material for: Patterns and predictors of language representation and the influence of epilepsy surgery on language reorganization in children and young adults with focal lesional epilepsy
Source: PLoS One. 2020 Sep 8;15(9):e0238389. doi: 10.1371/journal.pone.0238389 (PMC7478845; doi:10.1371/journal.pone.0238389)
Supplement: S2 Table — Univariate analysis. (DOCX) [file pone.0238389.s004.docx]

| **S2 Table. Generalized linear models. Univariate analysis** | | | | | | |
| --- | --- | --- | --- | --- | --- | --- |
|  | |  | **OR** | **P-value** | **LR χ^2^**  **(P-value)** | **Pseudo R^2^** |
| **IntraActWG** | | | | | | |
|  | SOZvsLe |  | 0.21 | 0.190 | 2.07 (0.150) | 0.055 |
|  | UnilobvsMultilobSOZ |  | 0.27 | 0.165 | 2.10 (0.147) | 0.056 |
|  | SideLe (Right vs Left) |  | 0.15 | 0.108 | 3.30 (0.070) | 0.088 |
|  | UnilobvsMultilobLe |  | 2.20 | 0.416 | 0.69 (0.406) | 0.018 |
|  | TLEvs ExT (TLE vs Fr) |  | 1.12 | 0.937 | 0.53 (0.768) | 0.014 |
|  | (TLE vs TPO) |  | 1.87 | 0.474 |  |  |
|  | SzFreq (NoSz vsWe) |  | 2.70 | 0.609 | 0.70 (0.704) | 0.020 |
|  | (NoSz vs Mo) |  | 4.15 | 0.427 |  |  |
|  | EpiDur |  | 1.13 | 0.135 | 2.54 (0.111) | 0.068 |
|  | Handed (Right vs Left) |  | 2.58 | 0.591 | 0.30 (0.582) | 0.008 |
|  | IctEEGfoc (Foc vs Diff) |  | 12.83 | **0.031** | 6.51 (0.011) | 0.188 |
|  | InterEEGfoc (Foc vs Diff) |  | 0.90 | 0.901 | 0.02 (0.901) | 0.000 |
|  | SzOnAge |  | 0.91 | 0.275 | 1.25 (0.263) | 0.033 |
|  | Histol (FCDI+FCDIIa vs FCDII) |  | 1.50 | 0.779 | 1.86 (0.601) | 0.052 |
|  | (FCDI+FCDIIa vs GNT+ FCDIIIb) |  | 0.42 | 0.407 |  |  |
|  | (FCDI+FCDIIa vs Other tumors + FCDIIIc + FCDIIId) |  | 0.33 | 0.383 |  |  |
| **NoPeriActWG** | | | | | | |
|  | SOZvsLe |  | 0.42 | 0.389 | 0.76 (0.383) | 0.015 |
|  | UnilobvsMultilobSOZ |  | 0.71 | 0.698 | 0.15 (0.698) | 0.003 |
|  | SideLe (Right vs Left) |  | 0.18 | 0.101 | 2.96 (0.086) | 0.058 |
|  | UnilobvsMultilobLe |  | 5.41 | 0.089 | 3.25 (0.072) | 0.064 |
|  | TLEvs ExT (TLE vs Fr) |  | 13.40 | 0.095 | 2.92 (0.233) | 0.058 |
|  | (TLE vs TPO) |  | 1.09 | 0.918 |  |  |
|  | SzFreq (NoSz vs Da) |  | 0.57 | 0.764 | 1.99 (0.738) | 0.039 |
|  | (NoSz vsWe) |  | 0.53 | 0.738 |  |  |
|  | (NoSz vs Mo) |  | 0.11 | 0.320 |  |  |
|  | (NoSz vs Ye) |  | 1.00 | 1.000 |  |  |
|  | EpiDur |  | 1.12 | 0.138 | 2.32 (0.128) | 0.046 |
|  | Handed (Right vs Left) |  | 0.75 | 0.799 | 0.06 (0.799) | 0.001 |
|  | IctEEGfoc (Foc vs Diff) |  | 6.27 | 0.050 | 4.47 (0.034) | 0.093 |
|  | InterEEGfoc (Foc vs Diff) |  | 2.68 | 0.265 | 1.29 (0.255) | 0.026 |
|  | SzOnAge |  | 0.97 | 0.703 | 0.15 (0.703) | 0.003 |
|  | Histol (FCDI+FCDIIa vs FCDII) |  | 0.17 | 0.224 | 6.64 (0.156) | 0.131 |
|  | (FCDI+FCDIIa vs GNT+ FCDIIIb) |  | 0.29 | 0.289 |  |  |
|  | (FCDI+FCDIIa vs Other tumors + FCDIIIc + FCDIIId) |  | 1.95 | 0.612 |  |  |
| **IntraActRG** | | | | | | |
|  | SOZvsLe |  | 0.67 | 0.712 | 0.14 (0.713) | 0.004 |
|  | UnilobvsMultilobSOZ |  | 0.28 | 0.289 | 1.31 (0.252) | 0.040 |
|  | SideLe (Right vs Left) |  | 0.25 | 0.166 | 2.05 (0.152) | 0.063 |
|  | UnilobvsMultilobLe |  | 0.28 | 0.289 | 1.31 (0.252) | 0.040 |
|  | TLEvs ExT (TLE vs TPO) |  | 0.56 | 0.562 | 0.35 (0.556) | 0.011 |
|  | SzFreq |  | Fisher's exact test p-value = 0.269* | | | |
|  | EpiDur |  | 1.25 | **0.049** | 5.65 (0.017) | 0.173 |
|  | Handed (Right vs Left) |  | 3.25 | 0.366 | 0.87 (0.350) | 0.027 |
|  | IctEEGfoc (Foc vs Diff) |  | 5.00 | 0.108 | 2.85 (0.092) | 0.094 |
|  | InterEEGfoc (Foc vs Diff) |  | 1.57 | 0.634 | 0.23 (0.634) | 0.007 |
|  | SzOnAge |  | 0.92 | 0.471 | 0.53 (0.466) | 0.016 |
|  | Histol (FCDI+FCDIIa vs FCDII) |  | 0.67 | 0.765 | 1.86 (0.602) | 0.057 |
|  | (FCDI+FCDIIa vs GNT + FCDIIIb) |  | 0.44 | 0.468 |  |  |
|  | (FCDI+FCDIIa vs Other tumors + FCDIIIc + FCDIIId) |  | 0.17 | 0.657 |  |  |
| **NoPeriActRG** | | | | | | |
|  | SOZvsLe |  | 0.47 | 0.497 | 0.47 (0.494) | 0.010 |
|  | UnilobvsMultilobSOZ |  | 0.96 | 0.967 | 0.00 (0.967) | 0.000 |
|  | SideLe (Right vs Left) |  | 0.19 | 0.115 | 2.72 (0.099) | 0.059 |
|  | UnilobvsMultilobLe |  | 2.24 | 0.419 | 0.67 (0.414) | 0.014 |
|  | TLEvs ExT |  | Fisher's exact test p-value=0.247* | | | |
|  | SzFreq |  | Fisher's exact test p-value=0.510* | | | |
|  | EpiDur |  | 1.20 | 0.053 | 4.55 (0.033) | 0.098 |
|  | Handed (Right vs Left) |  | 0.72 | 0.775 | 0.08 (0.775) | 0.002 |
|  | IctEEGfoc (Foc vs Diff) |  | 4.83 | 0.103 | 2.97 (0.085) | 0.069 |
|  | InterEEGfoc (Foc vs Diff) |  | 5.17 | 0.115 | 2.72 (0.099) | 0.059 |
|  | SzOnAge |  | 0.98 | 0.850 | 0.04 (0.850) | 0.001 |
|  | Histol (FCDI+FCDIIa vs FCDII) |  | 0.21 | 0.269 | 4.75 (0.191) | 0.103 |
|  | (FCDI+FCDIIa vs GNT + FCDIIIb) |  | 0.09 | 0.057 |  |  |
|  | (FCDI+FCDIIa vs Other tumors + FCDIIIc + FCDIIId) |  | 0.45 | 0.561 |  |  |
| **LangAct** | | | | | | |
|  | SOZvsLe |  | 0.42 | 0.464 | 0.60 (0.437) | 0.016 |
|  | UnilobvsMultilobSOZ |  | 0.83 | 0.833 | 0.04 (0.834) | 0.001 |
|  | SideLe (Right vs Left) |  | 0.19 | 0.152 | 2.65 (0.103) | 0.071 |
|  | UnilobvsMultilobLe |  | 4.15 | 0.221 | 1.85 (0.173) | 0.049 |
|  | TLEvs ExT (TLE vs TPO) |  | 1.06 | 0.946 | 0.00 (0.946) | 0.001 |
|  | SzFreq |  | Fisher's exact test p-value=0.309* | | | |
|  | EpiDur |  | 1.12 | 0.150 | 2.51 (0.113) | 0.067 |
|  | Handed (Right vs Left) |  | 0.47 | 0.488 | 0.47 (0.491) | 0.012 |
|  | IctEEGfoc (Foc vs Diff) |  | Fisher's exact test p-value=0.012* | | | |
|  | InterEEGfoc (Foc vs Diff) |  | 2.33 | 0.358 | 0.91 (0.340) | 0.024 |
|  | SzOnAge |  | 0.98 | 0.859 | 0.03 (0.858) | 0.001 |
|  | Histol (FCDI+FCDIIa vs FCDII) |  | 0.60 | 0.748 | 3.22 (0.359) | 0.091 |
|  | (FCDI+FCDIIa vs GNT + FCDIIIb) |  | 0.20 | 0.194 |  |  |
|  | (FCDI+FCDIIa vs Other tumors + FCDIIIc + FCDIIId) |  | 1.00 | 1.000 |  |  |
| **IntraActComp** | | | | | | |
|  | SOZvsLe |  | 1.22 | 0.855 | 0.03 (0.855) | 0.001 |
|  | UnilobvsMultilobSOZ |  | 0.50 | 0.478 | 0.51 (0.476) | 0.015 |
|  | SideLe (Right vs Left) |  | 0.50 | 0.482 | 0.51 (0.474) | 0.015 |
|  | UnilobvsMultilobLe |  | 2.00 | 0.482 | 0.51 (0.474) | 0.015 |
|  | TLEvsExT |  | Fisher's exact test p-value= 0.662* | | | |
|  | SzFreq |  | Fisher's exact test p-value= 0.591* | | | |
|  | EpiDur |  | 1.10 | 0.208 | 1.77 (0.183) | 0.054 |
|  | Handed (Right vs Left) |  | 0.81 | 0.855 | 0.03 (0.855) | 0.001 |
|  | IctEEGfoc (Foc vs Diff) |  | 5.40 | 0.088 | 3.25 (0.071) | 0.107 |
|  | InterEEGfoc (Foc vs Diff) |  | 0.78 | 0.772 | 0.08 (0.772) | 0.002 |
|  | SzOnAge |  | 0.99 | 0.906 | 0.01 (0.906) | 0.000 |
|  | Histol (FCDI+FCDIIa vs FCDII) |  | 0.50 | 0.661 | 0.63 (0.890) | 0.020 |
|  | (FCDI+FCDIIa vs GNT + FCDIIIb) |  | 0.50 | 0.615 |  |  |
|  | (FCDI+FCDIIa vs Other tumors + FCDIIIc + FCDIIId) |  | 1.00 | 1.000 |  |  |
| **PeriActComp** | | | | | | |
|  | SOZvsLe |  | 0.73 | 0.783 | 0.08 (0.783) | 0.002 |
|  | UnilobvsMultilobSOZ |  | 0.38 | 0.296 | 1.11 (0.291) | 0.024 |
|  | SideLe (Right vs Left) |  | 0.16 | 0.075 | 3.54 (0.060) | 0.077 |
|  | UnilobvsMultilobLe |  | 6.27 | 0.075 | 3.54 (0.060) | 0.077 |
|  | TLEvs ExT (TLE vs Fr) |  | 6.92 | 0.195 | 1.87 (0.393) | 0.041 |
|  | (TLE vs TPO) |  | 0.84 | 0.860 |  |  |
|  | SzFreq |  | Fisher's exact test p-value= 0.671* | | | |
|  | EpiDur |  | 1.02 | 0.781 | 0.08 (0.780) | 0.002 |
|  | Handed (Right vs Left) |  | 0.59 | 0.608 | 0.26 (0.608) | 0.006 |
|  | IctEEGfoc (Foc vs Diff) |  | 2.07 | 0.413 | 0.69 (0.407) | 0.017 |
|  | InterEEGfoc (Foc vs Diff) |  | 1.45 | 0.667 | 0.19 (0.666) | 0.004 |
|  | SzOnAge |  | 1.07 | 0.429 | 0.64 (0.425) | 0.014 |
|  | Histol |  | Fisher's exact test p-value= 0.059* | | | |
|  | | | | | | |
|  | |  | **Coeff.** | **P-value** | **LR χ^2^**  **(P-value)** | **Adjusted R^2^** |
| **LI-WG** | | | | | | |
|  | SOZvsLe |  | 0.31 | 0.189 | 1.90 (0.168) | 0.031 |
|  | UnilobvsMultilobSOZ |  | -0.24 | 0.261 | 1.39 (0.238) | 0.012 |
|  | SideLe (Right vs Left) |  | -0.27 | 0.237 | 1.54 (0.214) | 0.018 |
|  | UnilobvsMultilobLe |  | 0.20 | 0.369 | 0.89 (0.346) | -0.006 |
|  | TLEvs ExT (TLE vs Fr) |  | -0.06 | 0.870 | 0.49 (0.784) | -0.064 |
|  | (TLE vs TPO) |  | -0.14 | 0.516 |  |  |
|  | SzFreq (NoSZ vs Da) |  | -0.64 | 0.173 | 9.38 (0.052) | 0.165 |
|  | (NoSZ vs We) |  | -0.11 | 0.810 |  |  |
|  | (NoSZ vs Mo) |  | -0.25 | 0.629 |  |  |
|  | (NoSZ vs Ye) |  | -0.24 | 0.702 |  |  |
|  | EpiDur |  | -0.02 | 0.299 | 1.19 (0.275) | 0.005 |
|  | Handed (Right vs Left) |  | -0.94 | **0.001** | 13.3 (0.000) | 0.365 |
|  | IctEEGfoc (Foc vs Diff) |  | -0.15 | 0.488 | 0.53 (0.465) | -0.021 |
|  | InterEEGfoc (Foc vs Diff) |  | -0.55 | 0.004 | 9.14 (0.002) | 0.259 |
|  | SzOnAge |  | 0.04 | 0.056 | 4.02 (0.045) | 0.104 |
|  | Histol (FCDI+FCDIIa vs FCDII) |  | 0.01 | 0.969 | 0.70 (0.873) | -0.106 |
|  | (FCDI+FCDIIa vs GNT + FCDIIIb) |  | -0.13 | 0.529 |  |  |
|  | (FCDI+FCDIIa vs Other tumors + FCDIIIc +FCDIIId) |  | -0.05 | 0.856 |  |  |
| **LI-RG** | | | | | | |
|  | SOZvsLe |  | 0.28 | 0.175 | 2.07 (0.150) | 0.042 |
|  | UnilobvsMultilobSOZ |  | 0.12 | 0.521 | 0.46 (0.497) | -0.027 |
|  | SideLe (Right vs Left) |  | -0.04 | 0.805 | 0.07 (0.793) | -0.044 |
|  | UnilobvsMultilobLe |  | 0.17 | 0.310 | 1.15 (0.282) | 0.004 |
|  | TLEvs ExT (TLE vs Fr) |  | 0.27 | 0.421 | 2.87 (0.239) | 0.029 |
|  | (TLE vs TPO) |  | 0.25 | 0.152 |  |  |
|  | SzFreq (NoSZ vs Da) |  | -0.43 | 0.262 | 1.88 (0.758) | -0.126 |
|  | (NoSZ vs We) |  | -0.35 | 0.364 |  |  |
|  | (NoSZ vs Mo) |  | -0.46 | 0.306 |  |  |
|  | (NoSZ vs Ye) |  | -0.42 | 0.413 |  |  |
|  | EpiDur |  | 0.00 | 0.743 | 0.12 (0.729) | -0.042 |
|  | Handed (Right vs Left) |  | -0.28 | 0.181 | 2.00 ( 0.157) | 0.040 |
|  | IctEEGfoc (Foc vs Diff) |  | -0.05 | 0.736 | 0.13 (0.720) | -0.046 |
|  | InterEEGfoc (Foc vs Diff) |  | -0.08 | 0.629 | 0.26 (0.608) | -0.036 |
|  | SzOnAge |  | -0.01 | 0.780 | 0.09 (0.767) | -0.043 |
|  | Histol (FCDI+FCDIIa vs FCDII) |  | 0.29 | 0.217 | 2.43 (0.489) | -0.042 |
|  | (FCDI+FCDIIa vs GNT + FCDIIIb) |  | 0.02 | 0.909 |  |  |
|  | (FCDI+FCDIIa vs Other tumors + FCDIIIc + FCDIIId) |  | 0.11 | 0.625 |  |  |
| **LI-Comp** | | | | | | |
|  | SOZvsLe |  | -0.04 | 0.899 | 0.02 (0.893) | -0.045 |
|  | UnilobvsMultilobSOZ |  | -0.25 | 0.330 | 1.06 (0.304) | 0.000 |
|  | SideLe (Right vs Left) |  | 0.10 | 0.713 | 0.15 (0.698) | -0.039 |
|  | UnilobvsMultilobLe |  | 0.16 | 0.559 | 0.38 (0.537) | -0.029 |
|  | TLEvs ExT (TLE vs Fr) |  | -0.38 | 0.380 | 0.92 (0.630) | -0.054 |
|  | (TLE vs TPO) |  | -0.01 | 0.981 |  |  |
|  | SzFreq (NoSZ vs Da) |  | -0.27 | 0.642 | 4.38 (0.223) | 0.042 |
|  | (NoSZ vs Mo) |  | 0.14 | 0.815 |  |  |
|  | (NoSZ vs Ye) |  | 0.48 | 0.545 |  |  |
|  | EpiDur |  | -0.03 | 0.091 | 3.19 (0.074) | 0.085 |
|  | Handed (Right vs Left) |  | -0.69 | **0.020** | 6.02 (0.014) | 0.187 |
|  | IctEEGfoc (Foc vs Diff) |  | -0.08 | 0.767 | 0.10 (0.753) | -0.045 |
|  | InterEEGfoc (Foc vs Diff) |  | -0.24 | 0.335 | 1.04 (0.309) | -0.001 |
|  | SzOnAge |  | -0.05 | 0.036 | 4.93 (0.026) | 0.149 |
|  | Histol (FCDI+FCDIIa vs FCDII) |  | -0.33 | 0.414 | 0.91 (0.822) | -0.113 |
|  | (FCDI+FCDIIa vs GNT + FCDIIIb) |  | -0.14 | 0.682 |  |  |
|  | (FCDI+FCDIIa vs Other tumors + FCDIIIc+ FCDIIId) |  | -0.21 | 0.573 |  |  |

* When GLR estimates had standard errors questionable or the confidence intervals cannot be estimated, we reported the Fisher's exact test of contingency tables.

**The significant P-values are in bold.**

^a^ Coeff.=Regression coefficient; Comp: comprehension task; Da: daily; Diff: diffuse; EpiDur: Epilepsy duration; ExT: extratemporal; FCD: focal cortical dysplasia; Fr: Frontal; Foc: focal; GNT: glioneuronal tumors; Handed: handedness; Histol; histology; IctEEG: Ictal EEG, InterEEG: interictal EEG; IntraActComp: intralesional activation during comprehension task; IntraActRG: intralesional activation during RG task; IntraActWG: intralesional activation during WG task; LangAct: fMRI activation during all language tasks; Le: lesion; LI: laterality index; LR χ^2^ (P-value)= Likelihood Ratio test (p-value); Mo: monthly; Multilob: multilobar; NoPeriActRG: absence of perilesional fMRI activation during RG task; NoPeriActWG: absence of perilesional fMRI activation during WG task; NoSZ: no seizures at the assessment; OR=Odds Ratio; PeriActComp: perilesional fMRI activation during comprehension task; REF: reference; RG: rhyme generation, SzFreq: sizure frequency, SOZ: seizure onset zone; SzOnAge: age at seizure onset; TLE: temporal lobe epilepsy; TPO: temporo-parieto-occipital; Unilob: unilobar; VS: versus; Ye: yearly; We: weekly; WG: word generation.
