# Supplementary material for: Patterns and predictors of language representation and the influence of epilepsy surgery on language reorganization in children and young adults with focal lesional epilepsy
Source: PLoS One. 2020 Sep 8;15(9):e0238389. doi: 10.1371/journal.pone.0238389 (PMC7478845; doi:10.1371/journal.pone.0238389)
Supplement: S3 Table — (DOC) [file pone.0238389.s005.doc]

| **S3 Table: Postoperative analysis of associations: significant results** | | | | |
| --- | --- | --- | --- | --- |
| **Predictors** |  | **Outcomes** | |  |
|  |  | *Normal Post-operative EEG* | *Abnormal Postoperative EEG* | P-value |
| Global Delta-LI for RG task | | 0.09±0.16 | -0.40±0.34 | 0.003 |
| Temporal Delta-LI for RG task | | 0.23±0.38 | -0.30±0.54 | 0.047 |
| Front-Dorsal Delta-LI for RG task | | 0.06±0.20 | -0.48±0.38 | 0.004 |
|  |  |  |  |  |
|  |  | *Engel class Ia* | *Engel class IB-IV* | P-value |
|  |  |  |  |  |
| Global Delta-LI for WG task | | 0.05±0.32 | 0.48±0.58 | 0.015 |
| Front-Dorsal Delta-LI for RG task | | 0.05±0.33 | -0.47±0.55 | 0.046 |
|  |  |  |  |  |
|  |  | *Correlation with the disease duration* | | P-value |
| Global Delta-LI for RG task | | -0.660 |  | 0.005 |
| Temporal Delta-LI for RG task | | -0.596 |  | 0.019 |
