# Supplementary material for: Patterns and predictors of language representation and the influence of epilepsy surgery on language reorganization in children and young adults with focal lesional epilepsy
Source: PLoS One. 2020 Sep 8;15(9):e0238389. doi: 10.1371/journal.pone.0238389 (PMC7478845; doi:10.1371/journal.pone.0238389)
Supplement: S1 File — (DOCX) [file pone.0238389.s006.docx]

**Repository for MRI storage**

DOI

**10.12751/g-node.0vtcij**

URL

<https://doi.org/10.12751/g-node.0vtcij>.
